# Anxiety towards research and associated factors among postgraduate students of Jimma University Institute of Health, southwest Ethiopia

**DOI:** 10.1371/journal.pmen.0000646

**Published:** 2026-07-02

**Authors:** Diriba Kone Leta, Bekana Fekecha Hurissa, Gemechu Terefe Sori

**Affiliations:** School of Midwifery, Institute of Health, Jimma University, Jimma, Ethiopia; PLOS: Public Library of Science, UNITED KINGDOM OF GREAT BRITAIN AND NORTHERN IRELAND

## Abstract

Anxiety towards research among post-graduate students is an increasing concern, prominently affecting research productivity, academic performance and the overall mental health of the students. Therefore, this study aimed to assess levels of anxiety toward research and associated factors among postgraduate students at Jimma University Institute of Health, southwest Ethiopia. An institutional-based cross-sectional study was conducted from May 20 to June 20, 2024. The study was conducted among 422 postgraduate students selected by stratified random sampling method. Data was collected through a pretested online survey on Google forms. The collected data were checked, cleaned, and coded in Microsoft Excel and then exported to SPSS version 26 for analysis. A multivariable logistic regression analysis was conducted to analyze the association between dependent and independent variables. The associated factors were declared at p-value < 0.05 with AOR at 95% confidence interval (CI). From the total sampled students, 407(96.44%) were respondents in this study and 232 (57%; 95% CI: 52%, 62%) were reported to have a high research anxiety level. Being female (AOR = 6.54), those in MSc/MPH programs (AOR = 4.53), low research self-efficacy (AOR = 2.18), weak supervision quality (AOR = 2.76) and poor research infrastructure (AOR = 10.21) were significantly associated with high research anxiety level. This study shows a high research anxiety level among postgraduate students and factors such as demographic factors, research self-efficacy, supervision quality, and research infrastructure were significantly associated with student’s anxiety level. Providing targeted support for female students, enhancing self-efficacy, improving supervision quality, and strengthening research infrastructure, are essential for reducing research anxiety and promoting academic success.

## Introduction

In today’s dynamic world, the significance of research and scientific production (such as publications and innovations) as the benchmark for the advancement of nations and societies is unequivocal. Research has become a crucial intellectual asset that enables individuals to adapt to the evolving needs of society [1], shaping the world and facilitating new experiences [2]. Research as an essential tool for progress, is fundamental for significant advancements in both scientific and non-scientific domains [3]. Likewise, research is the backbone of the rapidly evolving field of medical sciences. In essence, research emerges as an indispensable force driving progress and shaping the future across diverse disciplines [4].

Scientific research employs systematic inquiry and empirical evidence to address real-world challenges, emphasizing rigorous methodology and ethical considerations throughout the process [5,6]. It aims to generate new knowledge, validate theories, and offer solutions by critically examining problems and reviewing existing literature [7,8].

Post-graduate(PG) education, extending beyond a bachelor’s degree, prioritizes research thesis and requires the completion of a research report as a partial requirement for earning PG degrees in higher education institutions [8,9]. PG training, particularly at the master’s and doctoral levels, builds a scientific, rational, and ethical foundation for future medical practices, with a significant focus on research activities that address existing literature gaps [3,7]. However, writing a research as a prerequisite for obtaining a degree poses considerable challenges for PG students, including limited research experience, methodological complexity, resource constraints and difficulties in academic writing [10].

At Jimma University Institute of Health (JUIH), PG students are required to complete research projects under time and resource constraints, and similar challenges related to limited research support and infrastructure have been reported in comparable settings [11]. However, the extent of research anxiety and its associated factors in this population has not been clearly understood.

Anxiety encompasses feelings of worry, dread, and unease [12]. It is the emotional stress experienced by individuals, particularly students, during research activities, stemming from apprehension about research tasks, fears of failure, uncertainties about methodologies, and academic expectations [8,13]. Research anxiety as a global phenomenon, creating disturbance and emotional distress among students around the world, and it is a widespread element throughout the research journey [14,15]. In recent years, the academic community has increasingly recognized that research significantly increases the likelihood of anxiety among PG students [16]. PG students are vulnerable to research anxiety and its detrimental impacts, due to academic pressure, uncertainty in the research process, and limited research experience [17].

The World Health Organization (WHO) highlights mental health as a critical component of overall well-being and academic success [18]. Similarly, the United Nations Educational, Scientific and Cultural Organization (UNESCO) underscores the role of higher education institutions in fostering supportive learning environments that promote both academic achievement and psychological well-being [19]. These priorities are further aligned with the United Nations Sustainable Development Goal 3 (SDG 3), which aims to promote good health and well-being, and Sustainable Development Goal 4 (SDG 4), which advocates for quality education [20]. The African Union Agenda 2063: The Africa We Want also emphasizes the need for a well-educated and skilled population supported by strong research and innovation systems to drive sustainable development across Africa [21].

Nevertheless, during the past two decades, there has been a relative shortage of research capacity and scientific output, particularly in low- and middle-income countries, creating a demand for more clinical and basic health science researchers [22–24]. A worrying trend is the prevalence and progressive increase in the pervasiveness of anxiety among PG students in the process of research production, emerging as a top concern within academic institutions [15]. This trend has far-reaching implications for the quality and productivity of the research process, extending beyond emotional distress, impacting both academic performance and the overall mental health of students [25].

If not effectively addressed, these anxieties may negatively impact the research process and the standard of research. Consequently, many PG students either discontinue their studies prematurely or fail to complete their PG degrees within the designated time frame [26]. For instance, approximately 50% of doctoral students in the United State of America (USA) fail to finish their degrees, dropping out during either the research proposal or dissertation-writing stages [27]. Some students choose to seek solutions and put effort into completing their required research writing by engaging in unethical practices in the research process. Consequently, they invest money in hiring another student to write their research papers for them [28].

They often fear being judged negatively for their writing competency, which leads to avoidance behavior whenever possible, and when they are required to write, they may demonstrate maladaptive writing behaviors [29], coupled with lower-than-average levels of research activity [1]. This points to a concerning trend of poor research quality and output, indicative of a substantial gap in research anxiety within the academic community [30].

Globally, PG students experience anxiety rates six times higher than the general population [31,32]. Research anxiety is prevalent among PG students due to factors such as inadequate training, external pressures, limited institutional support, limited exposure, resource constraints, and supervision quality [8,33–35]. This anxiety also stems from contributors such as research self-efficacy (RSE), publication expectations, time constraints, family support, individual traits and cultural influences [8,12,13,36–41]. Socio-demographic factors and systemic challenges, including economic issues and gender-related biases, also contribute to anxiety in PG students engagement in a research [3,9].

Unquestionably, elevated anxiety related to research writing among PG students can badly affect academic performance, leading to consequences such as reduced concentration and poor research output [8,42]. This anxiety impedes research productivity, limits academic achievements, and can discourage students from pursuing research-oriented careers, affecting the growth of the academic workforce [3,33]. Research writing anxiety needs serious attention, necessitating its measurement as an initial step toward identifying suitable strategies for research and thesis writing [42].

In Ethiopia, first-generation research-oriented universities exhibit a lag in publication numbers, reflecting a larger gap in research output among institutions [43]. The research community’s development ambition is impeded by inadequate funding, a lack of facilities, and a lack of interest in conducting research, [44], resulting in persistently low research output from the country’s higher education system, despite prioritization in policy documents and university mission and vision statements [43].

Limited studies explored anxiety toward research among PG students, focusing on factors such as RSE, infrastructure, socio-demographic influences, and cultural biases, and such evidence is especially scarce in settings like Ethiopia [12,38,39,45]. These investigations highlighted consequences ranging from compromised academic performance to limited research productivity. Despite interventions such as supervision programs and efforts to enhance research infrastructure, empirical studies on research anxiety are scarce [45]. To the best of investigator’s knowledge, there is limited published literature in Ethiopia specifically assessing the anxiety of PG students toward a research, although related studies have examined broader mental health issues such as stress [46]. Therefore, this research aims to fill this gap by assessing level of anxiety towards research and associated factors among PG students of JUIH, southwest Ethiopia and providing information on targeted interventions and contributing to a broader understanding of research anxiety.

## Methods and materials

### Ethics statement

Ethical approval was obtained from the JU institutional review board (IRB) with reference number (JUIH/IRB/071/24) of JU. Following this, an official letter was secured to get support from the head of each department involved in the study. After permission was obtained from each department the questionnaire was distributed to eligible students through official departmental communication channels, including Telegram, Imo, and Emails. To ensure that only eligible participants were included, the survey link was shared exclusively within PG student groups and networks affiliated with JUIH. In addition, a screening question was included at the beginning of the questionnaire to confirm that participants were currently enrolled PG students at JUIH and actively engaged in their research. Participants who did not meet these criteria were not allowed to proceed with the survey.

The students were asked to continue the survey once they had read the introduction of the questionnaire, including the purpose of the study, consent to participate, and the confidentiality issue as well as their right to discontinue even if they had started to fill it out. Participants were informed that their participation was voluntary, and confidentiality and anonymity were strictly maintained throughout the study. Personal information was not required on the questionnaire, ensuring that participants’ identities remained protected (See S1 Text).

### Study setting and period

The study was conducted from May 20 to June 20, 2024 at JUIH. JUIH is part of Jimma University (JU), a public higher education institution, located in Jimma, a city in the Oromia region in southern Ethiopia, which is 350 km from Addis Ababa. Founded in 1983, JU has grown into one of Ethiopia’s leading institutions, consistently striving for excellence in education, research, and community engagement. It is one of the universities designated as a research-intensive institution by the Ministry of Education in Ethiopia. JUIH, within this larger academic framework, focuses specifically on health-related disciplines, producing graduates who contribute to the improvement of healthcare services and the advancement of medical knowledge [47].

JUIH has three faculties; namely, Health Sciences, Medical sciences, and Public Health. These faculties encompass 22 departments, offer a range of academic programs, including undergraduate and graduate degrees in various health sciences disciplines, and hosts a diverse population of PG students. PG education at JUIH is designed to equip students with the knowledge and skills necessary to contribute meaningfully to their respective fields, and requires mandatory research thesis completion. Programs emphasize research as a cornerstone of academic and professional development. PG students participate in a rigorous curriculum that includes theoretical coursework, practical training, and research activities [48].

### Study design

An institutional-based cross-sectional study design was employed, as it allows for the assessment of the prevalence of research anxiety and its associated factors among PG students at a specific point in time.

### Study population and eligibility criteria

All sampled PG students who attended JUIH and were engaged in the pursuit of their PG research in the academic year 2024 were considered. Participants in this study were required to be enrolled in a PG program at JUIH during the study period, regardless of enrolment status (full-time or part-time), and actively engaged in their master’s or doctoral research to be included in the study. At JUIH, master’s programs typically have an expected duration of two years, while doctoral programs have a duration of four years. However, eligibility for this study was not based on program duration but rather on active engagement in the research process. Specifically, eligible participants had to have established a research title, been assigned a research supervisor, and either begun data collection or initiated data analysis at the time of data collection. Students were excluded from the study if they were unable to provide their information due to illness, or inaccessibility.

### Sample size and sampling procedures

The sample size for this study was determined using a single population formula, with a conservative assumption of 50% prevalence for both the dependent and independent variables. Since no similar study was available from the study area or within the Ethiopian context, the 50% prevalence was used as a rule of thumb to ensure sufficient power for the study. To calculate the sample size, the following formula was used:


$$\begin{array}{c} 𝐧\;=\;\frac{{\left({𝐙}_{\frac{\alpha }{2}}\right)}^{2}𝐩\left(1-𝐩\right)}{{𝐝}^{2}} \end{array}$$

**Where:** **n** is the minimum sample size required

**Z**_**α/2**_ is the standard score value for the 95% confidence level (1.96)

**p** is the estimated prevalence (50%)

**d** is the margin of error (5%)

Therefore, the sample size of 384 was initially determined. After adding a 10% non-response rate, the final sample size was adjusted to 422, representing 74% of the target population. According to evidence obtained from the Jimma University Registrar’s Office and each department under JUIH, there were a total number of 573 PG students engaged in research during this study period. A sampling frame was developed accordingly based on this list of eligible students. Since the source population was finite, the finite population correction was calculated, yielding a corrected sample size of 243. However, the initially calculated sample size was retained to increase the precision of the estimates and ensure adequate statistical power.

This study utilized a stratified random sampling technique, which involved several key steps to ensure a representative sample. Initially, stratification was done based on program level, categorizing students into Master of Science (MSc)/Master of Public Health (MPH), specialty, sub-specialty, and Doctor of philosophy (PhD) programs. Within each of these strata, further stratification was performed according to individual disciplines to ensure that all disciplines were adequately represented. The total number of eligible students in each stratum was obtained from institutional records, and proportional allocation was applied (See S2 Table). A random sample of PG students was then selected from each stratum, with these individuals serving as the primary sampling units. This methodology ensured that every PG student within each discipline had an equal opportunity to be included in the study, thereby enhancing the representativeness of the sample.

### Conceptual definition and operational definition of terms

**Research anxiety**: is conceptualized as a form of situational anxiety characterized by feelings of apprehension, tension, and worry experienced when engaging in research related tasks. This aligns with established psychological frameworks that define anxiety as a future-oriented emotional state involving cognitive, affective, and physiological responses to perceived demands or threats [49,50].

**Research anxiety level**: refers to the quantifiable degree of apprehension, unease, or nervousness experienced by PG students in response to engaging in research activities. It was measured by using a set of 4 items rated on a 7-point Likert scale ranging from 1 (strongly disagree) to 7 (strongly agree). To create a composite measure of research anxiety, the average score across the 4 items is calculated for each participant, with possible scores ranging from 1 to 7. The average score is then used to categorize participants into two distinct groups based on the median value of the composite scores, as the data was skewed based on the distribution of responses to anxiety-related statements (See S4 Table). Participants who scored greater than or equals to the median were classified as experiencing high anxiety towards their research, while those scoring below the median were categorized as having low research anxiety level [12,35,51].

**Self-efficacy in research**: refers to the belief of an individual’s in their competence and abilities to successfully perform tasks related to research activities, consistent with social cognitive theory, which emphasizes self-efficacy as a determinant of behavior and performance [52,53]. It was measured using 18-item instrument, with each item rated on a 7-point Likert scale ranging from 1 (strongly disagree) to 7 (strongly agree). To obtain a composite measure of RSE, the average score across the 18 items is calculated for each participant. Participants are then categorized into two groups based on the median value of these composite scores. Those with scores greater than the median were classified as having high research self-efficacy, while those with scores less than or equal to the median were considered to have low research self-efficacy [37,54].

**Quality of supervision:** refers to the perceived effectiveness, support, mentorship, and guidance provided by the supervisors to PG students in the context of their research activities [55]. It was assessed through 6 items rated on a 5-point Likert scale, ranging from 1 (strongly disagree) to 5 (strongly agree). A composite measure was obtained by calculating the average score across these 6 items for each participant, with possible scores ranging from 1 to 5. Participants were then divided into two groups based on the median value of these scores. Those scoring greater than the median were categorized as perceiving strong supervision quality, whereas those scoring less than or equal to the median were categorized as perceiving weak supervision quality [9,56,57].

**Research infrastructure:** refers to the adequacy and availability of physical, institutional, and technical resources that support the conduct of research activities [58]. It was measured using 5 items rated on a 5-point Likert scale, from 1 (strongly disagree) to 5 (strongly agree). The average score across these 5 items was calculated for each participant to create a composite measure. Participants were then categorized into two groups based on the median value of these scores. Those with scores greater than the median were classified as perceiving good research infrastructure, while those with scores less than or equal to the median were considered to perceive poor research infrastructure [57,59].

**Academic support**: refers to the emotional, informational and practical assistance provided by instructors, peers and family that facilitates students’ academic and research engagement [60,61]. It was measured using a 12-item scale, each rated on a 5-point likert scale (1 = strongly disagree, 5 = strongly agree). The final academic support score for each student was derived by averaging the ratings across the 12 items. Those students scoring above the median were classified as receiving good academic support, whereas those scoring at or below the median were categorized as receiving poor academic support [9,62].

### Data collection tools and procedures

A self-administered, validated online survey instrument was utilized for data collection. The survey comprised a total of 49 elements: 4 items adapted from the revised scale of students’ attitudes toward research (R-SAR) for measuring research anxiety levels [51], 18 items adapted from the development of the research self-efficacy scale to measure the RSE of PG students [54], 6 items adapted from the Postgraduate Research Experience Questionnaire (PREQ) [57], 5 items adapted from the Postgraduate Research Experience Questionnaire (PREQ) [57], 12 items adapted from the perceived academic support questionnaire (PASQ) to assess academic support [62], and 4 items developed by the investigator to gather data on demographic information of the study participants (See S2 Text).

The adaptation of instruments was carried out by selecting relevant domains consistent with the study objectives. Specifically, only the anxiety related items from the R-SAR were used to measure research anxiety. Where applicable, the original sources were appropriately acknowledged.

The internal consistency of the adapted scales was assessed using Cronbach’s alpha, and all constructs demonstrated acceptable reliability, Cronbach’s alpha > 0.70 (See S1 Table). In addition, the instruments were reviewed for contextual relevance and clarity. Before the actual data collection, the survey instruments underwent a pretest with a small sample (5%) of PG students at Ambo University, two weeks in advance. Based on the pretest findings, minor modification were made to improve clarity and comprehension, including rephrasing ambiguous questions, and refining wording for better understanding of the questionnaire. Participants were provided with detailed information about the study, and an electronic written informed consent was obtained from all participants.

### Data quality assurance

Ensuring data quality is paramount for the validity and reliability of study findings. Several measures were implemented to safeguard data quality throughout the research process, including using standardized and validated tools, pretesting of instruments, and continuous monitoring. All questionnaire items were set as mandatory in the Google Forms platform to minimize missing data and ensure completeness of responses. The collected data was checked daily for completeness and consistency at the end of each data collection day. Data quality was further ensured by coding for missing values before data analysis. The reliability of the data collection instrument was assessed and the Cronbach’s alpha >0.7 deemed acceptable. Internal consistency was evaluated separately for each domain, and all domains demonstrated acceptable reliability (See S1 Table). The overall scale’s internal consistency coefficient was found to be 0.92 in this study. The estimated time required to complete the questionnaire was approximately 15 – 20 minutes, based on the pretest findings.

### Data processing and analysis

The collected quantitative data were checked, and cleaned in Microsoft Excel (See S1 Data). After coding, the data were exported to statistical package for the social sciences (SPSS) version 26 for analysis. Descriptive statistics, such as frequencies, percentages, means, medians, and standard deviations, were computed to summarize the findings. Binary logistic regression analysis was performed to identify variables with a p-value less than or equal to 0.25, which were considered for inclusion in the multivariable logistic regression model.

A multivariable logistic regression analysis was then conducted to examine the associations between dependent and independent variables. Associations were deemed significant with a p-value less than 0.05 and reported with an adjusted odds ratio (AOR) and a 95% confidence interval (CI). The model’s fitness was assessed using Hosmer and Lemeshow’s test, and multicollinearity was checked. The Hosmer and Lemesshow Test was 0.79 and Nagel kerke, R Square 0.57. All candidates for final model had variance inflection factor less than 1.5 and tolerance greater than 0.6. Therefore there were no issues of collinearity (See S3 Table). The results of the analysis were presented in tables, figures, and text, as appropriate.

## Results

### Demographic characteristics of respondents

From the total of 422 sampled students, 407(96.44%) were respondents in this study. The remaining 15 students did not respond to the survey invitation despite repeated remainders. From the different program level, majority were MSc/MPH 278(68.3%). Regarding their discipline the largest groups of participants were Clinical midwifery 15(3.7%), Clinical Pharmacy 15(3.7%), Integrated Clinical and Community Mental Health 15(3.7%), and Anesthesiology, Critical Care and Pain Medicine 15(3.7%) students. Among all respondents the majority, 327(80.3%) were males (Table 1). The median age of participants was 32 with an age range spanning from 24 to 44 years.

**Table 1:** Demographic characteristics of PG students of Jimma university institute of health, 2024 (N = 407).

Variable	Category	Frequency	Percent
Gender	Male	327	80.3
	Female	80	19.7
Program level	MSc/MPH	278	68.3
	Speciality	90	22.1
	Sub Speciality	10	2.5
	PhD	29	7.1
Discipline	Applied Ecology	3	.7
	Environmental Health	7	1.7
	Evidence Based Health Care	2	.5
	Health communication and Health Behavior	7	1.7
	Health System and Policy	4	1.0
	Human Nutrition	12	2.9
	Medical Microbiology	13	3.2
	Medical Physiology	9	2.2
	Pharmaceutical Sciences	1	.2
	Public Health - Reproductive Health	1	.2
	Tropical and Infectious Diseases	3	.7
	Adult health nursing	8	2.0
	Bio-informatics	4	1.0
	Child and Pediatrics Nursing	7	1.7
	Clinical Anesthesia	13	3.2
	Clinical Anatomy	4	1.0
	Clinical Laboratory Sciences Specialty in Clinical Chemistry	10	2.5
	Clinical Laboratory Sciences Specialty in Hematology and Immunohematology	5	1.2
	Clinical Laboratory Sciences Specialty Laboratory Management	4	1.0
	Clinical midwifery	15	3.7
	Clinical Pharmacy	15	3.7
	Dietetics	5	1.2
	Emergency critical care nursing	7	1.7
	Environmental health science and technology	8	2.0
	Epidemiology	14	3.4
	General Public Health	7	1.7
	Health Economics	7	1.7
	Health monitoring and evaluation	12	2.9
	Health Promotion and Health Behavior	5	1.2
	Health system management	7	1.7
	Integrated Clinical and Community Mental Health	15	3.7
	Maternity health nursing	9	2.2
	Medical Biochemistry	3	.7
	Medical Parasitology	7	1.7
	Midwifery Education	5	1.2
	Neonatal Health Nursing	5	1.2
	Pharmaceutical Quality Assurance and Regulatory Affairs	10	2.5
	Pharmaceutical supply Chain management	13	3.2
	Pharmaceutics	6	1.5
	Pharmacology	4	1.0
	Psychiatric and Mental Health Nursing	4	1.0
	Reproductive Health	7	1.7
	Anesthesiology, Critical Care And Pain Medicine	15	3.7
	Emergency and Critical Care Medicine	10	2.5
	Internal medicine	10	2.5
	Obstetrics and gynecology	12	2.9
	Ophthalmology	11	2.7
	Pediatrics and Child Health	8	2.0
	Pathology	3	.7
	Psychiatry	5	1.2
	Radiology	4	1.0
	Surgery	12	2.9
	Gastrointestinal Cancer Surgery	2	.5
	Gynecological Oncology	4	1.0
	Pediatrics Hematology Oncology	2	.5
	Urogynaecology	2	.5

### Other characteristics of the respondents

Out of the 407 participants in the study nearly half of the participants, 201 (49.4%) reported low RSE. Approximately 212(52.1%) of students reported that they received strong supervision. A majority of students 225(55.3%) rated the research infrastructure as ‘good’, while the remaining 182(44.7%) perceived the research infrastructure as ‘poor’ (Table 2).

**Table 2:** Characteristics factors of PG students of Jimma university institute of health, 2024 (N = 407).

Factor	Classification	Frequency	Percent
**Self-Efficacy in Research**	Low	201	49.4
	High	206	50.6
**Quality of the Supervision**	Weak	195	47.9
	Strong	212	52.1
**Research Infrastructure**	Poor	182	44.7
	Good	225	55.3
**Academic Support**	Poor	195	47.9
	Good	212	52.1

#### Anxiety level of PG students toward research at Jimma University Institute of Health.

**Fig 1:**
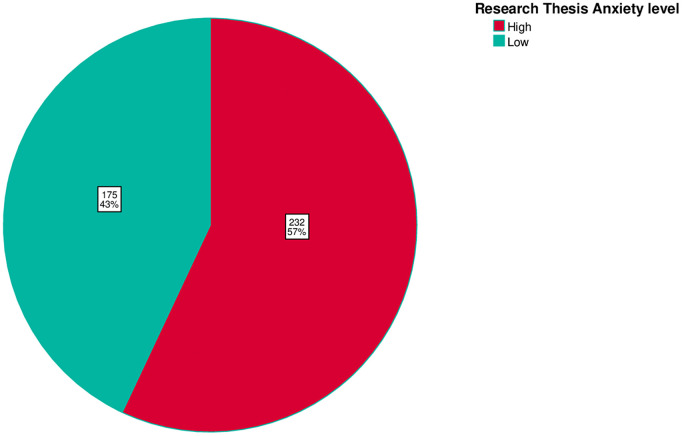
Research anxiety level of study participants among PG students at Jimma University Institute of Health, 2024 (N = 407).

Out of the 407 participants in this study, 232 (57%) students reported experiencing high anxiety regarding their research (Fig 1). Research anxiety levels varied significantly across different categories. Among female participants, 71 (88.75%) students reported high anxiety, while 161(49.24%) of male students reported high anxiety. Regarding program level, MSc/MPH students were the most affected, with 168 (60.4%) students reporting high anxiety (Table 3).

**Table 3:** Binary and Multivariable analysis to identify the factors associated with the anxiety level of PG students towards research at Jimma University Institute of Health, 2024 (n = 407).

Variable	Categories	Anxiety level		Binary analysis		Multivariable analysis	
		High N (%)	Low N (%)	COR and 95% CI	P value	AOR and 95% CI	P valve
Gender	Female	71(88.75)	9(11.25)	8.13(3.93,16.82)	.p < 0.001	6.54 (2.65, 16.12)	p < 0.001 **
	Male	161(49.24)	166(50.76)		1		1
Program level	MSc/MPH	168(60.4)	110(39.6)	5.86(2.31,14.84)	p < 0.001	4.53(1.29, 15.89)	.018 **
	Specialty	52(57.8)	38(42.2)	5.25(1.95,14.13)	.001 *	1.12(.29, 4.33)	.866
	Sub specialty	6(60)	4(40)	5.75(1.22,27.14)	.027 *	4.30(.68, 27.24)	.121
	PhD	6(20.7)	23(79.3)		1		
Research Self efficacy	Low	153(76.1)	48(23.9)	5.12(3.34, 7.87)	p < 0.001*	2.18(1.14, 4.17)	.019 **
	High	79(38.3)	127(61.7)		1		1
Supervision Quality	Weak	149(76.4)	46(23.6)	5.03(3.27, 7.74)	p < 0.001*	2.76(1.51, 5.06)	.001 **
	Strong	83(39.2)	129(60.8)		1		1
Research Infrastructure	Poor	156(85.7)	26(14.3)	11.76(7.14, 19.37)	p < 0.001*	10.21(5.58, 18.69)	p < 0.001**
	Good	76(33.8)	149(66.2)		1		
Academic Support	Poor	139(71.3)	56(28.7)	3.18(2.10, 4.80	p < 0.001*	1.08(.57, 2.04)	.819
	Good	93(43.9)	119(56.1)		1		

“*” Indicates factors associated with high research anxiety level in bivariable analysis, P value < 0.25,

“**” Indicates factors associated high research anxiety level in multivariable analysis, p value < 0.05,

1 = reference category.

Of the 201 students with low self-efficacy, 153 (76.1%) students reported high anxiety. One hundred forty-nine (76.4%) of Students who reported weak supervision experienced significantly higher levels of anxiety. Students who rated the research infrastructure as poor also reported significantly higher anxiety, with 156 (85.7%) students in this group experiencing high anxiety (Table 3).

#### Factors associated with the research anxiety level of PG students at Iimma University Institute of Health.

In bivariate logistic regression analysis factors such as gender (AOR 8.13, 95% CI (3.93,16.82), program level (AOR 5.85(2.31,14.84), research self-efficacy (AOR 5.12, 95% CI (3.34, 7.87), supervision quality (AOR 5.03, 95% CI (3.27, 7.74), research infrastructure (AOR 11.76, 95% CI (7.14, 19.37), and academic support (AOR 3.18, 95% CI (2.10, 4.80) were significantly associated with high anxiety levels among students at p- value ≤ 0.25 (Table 3). So, they were candidates for multi-variable logistic regression analysis.

A multivariable logistic regression analysis was used to retain factors at a significance level of P value less than 0.05. In multivariable logistic regression analysis, being females, those in MSc/MPH programs, having lower RSE, poor supervision quality, and inadequate research infrastructure were more likely to experience high anxiety. The odds of high research anxiety level was found to be more than six times higher in females than in males, AOR 6.54 (95% CI: 2.65, 16.12). Likewise the odds of high research anxiety were four and half times higher among MSc/MPH students than PhD students ((AOR = 4.53, 95% CI:(1.29, 15.89) (Table 3).

Additionally, students with low RSE were found to be 2.18 times more likely to report high anxiety compared to those with high self-efficacy (AOR = 2.18, 95% CI (1.14, 4.17). Similarly, the odds of high thesis anxiety were almost three times higher among participants with weak supervision quality than participants with strong supervision (AOR = 2.76, 95%CI (1.51, 5.06) and Lastly, the odds of high research anxiety were 10.21 times higher among participants with poor research infrastructure than participants with good infrastructure (AOR = 10.21, 95%CI (5.58, 18.69) (Table 3).

## Discussion

The finding of this study revealed that the magnitude of high research anxiety is 57% with 95% CI (52%, 62%) in JUIH. Regarding factors associated with level of anxiety towards research, this study finding revealed that being females, those in MSc/MPH programs, having lower RSE, with poor supervision quality, and inadequate research infrastructure were more likely to experience high anxiety.

The overall prevalence of research anxiety in this study aligns with results from other studies, such as those conducted in the Peru (85%), India (72.67%), United Kingdom (UK) (40.7%), China (20%), and Pakistan, where PG students consistently report heightened levels of anxiety related to research activities [2,35,63–65].

The finding of this study was higher than studies done in UK and China. The discrepancy of these findings can be attributed to different factors. The relatively lower prevalence in these high-income countries could be attributed to better research infrastructure, more supportive supervision, and greater access to mental health resources in these settings [63,64]. In comparison, the 57% magnitude of high anxiety found in this study suggests that Ethiopian students may experience more due to systemic challenges, within the academic environment, such as limited resources and less robust student support services [44]. Students from low income countries, like Ethiopia, face significant barriers, including inadequate access to libraries, research materials, and technological tools, all of which contribute to heightened stress and anxiety [37]. The lack of a robust support system exacerbates feelings of helplessness and frustration among students. Additionally, socioeconomic factors play a crucial role in increasing research anxiety. Many Ethiopian students experience financial difficulties, limiting their ability to access necessary resources like data collection tools or research materials [44]. These financial constraints, often compounded by family obligations or part-time jobs, contribute to higher stress levels. As students balance academic responsibilities with financial hardships, their anxiety increases [66].

Furthermore, the other possible reason of the discrepancy between finding of this study and studies in UK and China can be due to the difference in tool employed to measure anxiety. Data was collected by Generalized Anxiety Disorder-7 (GAD-7) for both studies [63,64], which contradicts to our study, which used items from the R-SAR for measuring research anxiety levels.

But, the finding of current study is lower than studies done in Peru and India [2,35]. The study done in Peru reported increased prevalence of anxiety and it might be partly due to the fact that the demanding nature of research programs in the region, where students face significant academic pressures with limited institutional support. It was a Probabilistic sample of 206 students and the instruments used in the research were the State-trait Anxiety inventory (STAI) [35], while our study utilized a stratified random sampling technique to select 407 participants. Our study and study done in India used the same tool to measure research anxiety level, however, study conducted in India purposely selected a small sample size (100 students) from single master of physiotherapy students [2], unlikely to our study which recruited several health and medical related disciplines from different PG program level including masters and PhD students. This study highlights the urgent need for targeted interventions to alleviate research anxiety among Ethiopian students.

From the result of this study, female students were over six and a half times more likely to experience research anxiety than male students. This heightened anxiety among females is consistent with findings from studies in the UK, China, Pakistan, Iran, and Turkey [28,63–65,67]. The reason might be due to the females are more emotionally perceived about anxiety than males [68,69]. Another reason might be due to their hormonal variation from males and their thoughts about their social situation [68]. Also female students mostly report experiences of inequality, discrimination, marginalization and silencing, which, in combination with other stressors related to PG research, may contribute to the vulnerability of female. This experienced discrimination can cause significant stress and increase the risk of poorer physical and mental health [70]

The elevated anxiety levels in female students may be linked to societal and cultural expectations that require them to balance academic responsibilities with family and caregiving roles. In Ethiopia and similar sociocultural contexts, these dual pressures can lead to feelings of overwhelm and increased stress during the research process [71]. Studies from Nigeria have shown that female PG students experience stress related to academic pressure, work-family conflict, and role expectations [72]. Also studies in the UK and China further emphasize that female PG students face higher anxiety due to these compounded academic and social demands [16,63,70]. These findings align with role strain theory, which posits that competing social roles can lead to increased stress when individuals lack sufficient resource to manage them [73]

Conversely, male students tend to report lower anxiety levels, possibly due to the absence of such societal pressures or because of greater societal acceptance of male students’ roles as full-time academics without the added responsibility of family obligations [65,67] On the other hand, male students may experience less social pressure, which could contribute to their lower anxiety levels. Men may also be less likely to express vulnerability or anxiety, leading to under reporting of such feelings. It is also possible that societal expectations of male students in academic settings allow them to approach their research with more confidence, reducing their anxiety levels in comparison to female students [70]. These findings underscore the need for gender-sensitive support in academic settings.

Likewise, program level was significantly associated and master’s students were approximately four and a half times more likely to report anxiety than those pursuing a PhD. This increased anxiety among MSc/MPH students is due to their limited research experience, shorter program duration, and the intense pressure to complete their thesis within a tight time frame [12,37]. This aligns with previous studies in countries like India, China, and Iran, which found that MSc students tend to report higher levels of anxiety due to these challenges [9,12,37,64]. In contrast, PhD students generally report lower anxiety levels, likely because of their advanced research skills, longer engagement with research, and more comprehensive guidance throughout their program. The extended timeline for PhD students allows for more thorough research refinement and support, reducing the pressure compared to the condensed schedule faced by MSc/MPH students [9,25,63].

Additionally, finding of this study revealed that low RSE significantly increases research anxiety among PG students. Those with lower confidence in their research abilities experience higher anxiety due to doubts about managing complex tasks and meeting academic standards. The lack of confidence leads to feelings of inadequacy, procrastination, and stress [74]. The present study’s findings align with previous studies in Iran, China, and India, which consistently show a negative association between research self-efficacy and anxiety [12,37,63,64]

A study conducted by Nazari et al. in Iran found that students who doubted their research abilities were more likely to struggle with feelings of uncertainty, fear of failure, and a lack of control over the research process, leading to increased anxiety [12]. In a study conducted in China, researchers concluded that students who believed in their ability to conduct research were less likely to experience anxiety, as they felt more capable of handling the demands of their research projects [64]. Similarly, a study in India also found that self-efficacy training could improve students’ confidence and reduce their anxiety, thus helping them cope better with the research process [37]. Furthermore, research conducted by Duncan and colleagues in the UK found that students who had a strong belief in their research abilities were better able to manage the stress and anxiety that typically accompany thesis writing. These findings aligns with the results of the present study and underscores the crucial role of RSE to manage research anxiety [63]. The inverse relationship between RSE and research anxiety observed in this study is strongly supported by social cognitive theory, which emphasizes the role of self-efficacy in shaping emotional responses to challenging tasks [75].

Furthermore, in this study, weak supervision increased the likelihood of research anxiety by nearly three times among PG students. This might be due to students who perceived their supervisors as unresponsive, inaccessible, or lacking in guidance were more likely to experience feelings of confusion, stress, and uncertain about quality of their research work. The lack of necessary support and timely and constructive feedback from the supervisor can exacerbate feelings of inadequacy and fear of failure, which in turn intensifies anxiety [56,64].

The finding of present study align with other studies, such as the one from China, India, the UK, and Pakistan, which consistently show that high-quality supervision, marked by regular feedback and strong support, correlates with lower research anxiety. In contrast, minimal supervision or feedback leads to students feeling overwhelmed and underprepared, escalating anxiety levels. Studies highlight that good supervision fosters confidence in the research process, while poor supervision results in heightened anxiety due to lack of clarity and direction [56,63,64]. This implies the importance of effective supervision in reducing research anxiety. Positive supervisor-student relationships help mitigate anxiety by offering clear feedback, guidance, and trust. Conversely, poor supervision can lead to feelings of isolation and uncertainty [12,63,65]. Supervisor’s clear expectations and regular feedback can help students feel more confident and reduce uncertainty [5].

Lastly, this study found that Students facing poor research infrastructure were over ten times more likely to experience high research anxiety compared to their counterparts, highlighting a strongly significant resource gap in JUIH where research infrastructure may be limited due to under-funding [44]. Inadequate infrastructure can create obstacles and roadblocks in completing the research tasks, leading to feelings of frustration, stress, and anxiety, particularly if students perceive that they lack the necessary resources to conduct their research effectively [76].

In consistent with our finding, studies from China, India, Pakistan, and the UK have confirmed that access to quality research resources is essential for reducing stress, enhancing confidence, and supporting students’ academic progress [37,63,64]. A study in China found that students who reported insufficient access to research facilities and outdated resources experienced higher levels of anxiety, as they struggled to carry out essential research tasks due to the lack of adequate support [76]. Similarly, a study from in India found that students who faced challenges in accessing research tools and resources were more likely to experience stress and frustration, leading to higher anxiety levels [12].

In Pakistan, a study also highlighted that students with limited access to research labs, specialized equipment, or databases were more likely to report feelings of uncertainty and stress. The lack of access to these resources left students struggling to complete their research tasks, which further contributed to anxiety [65]. A study in the UK also indicated that students, who had limited access to research tools, were more likely to experience anxiety, particularly when they felt that these limitations hindered their research progress [63]. The implication of these studies is that research infrastructure is a key factor in mitigating research anxiety

### Significance and practical implications of the study

The findings of this study offer valuable information on PG research challenges and contributing to the existing literature. The findings have broader implications for students, faculties, institutions, and the advancement of PG education and research. The findings of the study may also help provide baseline data for any future researchers interested in conducting further investigations on research anxiety.

### Strengths and limitations of the study

This study offers significant strengths, including a large sample size of 422 (74% of the target population) PG students from JUIH and sufficient response rate, 407 (96.44%), which enhances the generalizability of the findings. The use of a stratified random sampling technique ensured diverse representation across program levels and disciplines, while validated instruments and multivariable logistic regression analysis provided a rigorous framework for assessing anxiety levels and identifying associated factors.

It has also limitations that future research can address by building up on useful insights provided by current study. Its cross-sectional design restricts the ability to establish causal relationships between research anxiety and the identified predictors, and reliance on self-reported measures may introduce bias such as social desirability and recall biases. Additionally, the findings may not be fully generalizable to other institutions or cultural contexts, particularly given that the sample was predominantly composed of MSc/MPH students and males.

## Conclusion

This study reveals a high level of anxiety towards research among PG students at JUIH, compared with studies done in many high-income countries, indicating that unique challenges within the Ethiopian academic context contribute significantly to research anxiety. Key factors associated with this anxiety include demographic factors (gender and program level), research self-efficacy, supervision quality, and research infrastructure. Notably, females, those in MSc/MPH programs, low RSE, weak supervision quality, and poor research infrastructure reported higher research anxiety levels. The findings support relevant policy frameworks, such as SDG 3, SDG 4, and the African Union’s Agenda 2063. Research anxiety among PG students is real, and requires immediate attention from stakeholders. Targeted interventions, such as empowering female students in research activities, enhancing self-efficacy, improving supervision quality, and strengthening research infrastructure, are essential to mitigate the impact of this research anxiety and support students effectively.

## Supporting information

S1 Text Participant Information Sheet and informed consent form. (DOCX)

S2 Text Questionnaire. (DOCX)

S1 Data Postgraduate student's research anxiety and attitude data. (XLSX)

S1 Table Reliability analysis of the tools. (DOCX)

S2 Table Sample size allocation for program levels and disciplines. (DOCX)

S3 Table Multicollinearity assessment. (DOCX)

S4 Table Average, median and SD of the variables. (DOCX)
