# Supplementary material for: Anxiety towards research and associated factors among postgraduate students of Jimma University Institute of Health, southwest Ethiopia
Source: PLOS Ment Health. 2026 Jul 2;3(7):e0000646. doi: 10.1371/journal.pmen.0000646 (PMC13327115; doi:10.1371/journal.pmen.0000646)
Supplement: S1 Text — (DOCX) [file pmen.0000646.s001.docx]

**Participant Information Sheet and Informed Consent Form**

**Informed consent form**

Dear Participant, Greeting!

My name is Diriba Kone Leta. I am doing this research for the partial fulfillment of the requirements for a Master of Science degree in Midwifery Education. The purpose of the study is to assess the anxiety towards research among PG students and its associated factors within JUIH, southwest Ethiopia. To collect data, I invite you to take part in our research. Your cooperation and honest participation in filling out questionnaires will provide me valid result and help me to make recommendation of different type of relevant intervention. If you are willing, you need to understand and sign the consent form. By participating in this research, you may feel some discomfort in sacrificing your time otherwise, no risk in participating in this research, so your response provides an important input to show the gap and is essential for identifying targeted interventions to cultivate a robust and supportive research culture.

If you are participating in this research, the output of the study will have both direct and indirect benefits to you, as you and your family will benefit from the development of the next generation of scholars and researchers as an academic community in particular and as members of society in general. You will not be provided an incentive or payment to take part in this research. The information that we collect from this research will be kept private. Any information about you will have a number on it instead of your name. Only the researcher will know what your number is and we will use password protection. It will not be shared with or given to anyone except the data collector. You have the full right to refuse as well as withdraw from participating in this research. Your refusal will not affect you in any way.

**Informed consent form**

I have read and I understand the provided information and have had the opportunity to ask questions. I understand that my participation is voluntary and that I am free to withdraw at any time, without giving a reason and without cost. I voluntarily agree to take part in this study.

**Contact Information**

If you want to know more information at any time about this study, please contact the Principal Investigator through the following address. [Diriba Kone Leta: Tell: +251910964455 or email address [diribakone@gmail.com](mailto:diribakone@gmail.com)]

Participant's signature_____________________________ Date __________

Investigator's signature _____________________________ Date __________
