# Supplementary material for: Anxiety towards research and associated factors among postgraduate students of Jimma University Institute of Health, southwest Ethiopia
Source: PLOS Ment Health. 2026 Jul 2;3(7):e0000646. doi: 10.1371/journal.pmen.0000646 (PMC13327115; doi:10.1371/journal.pmen.0000646)
Supplement: S2 Text — (DOCX) [file pmen.0000646.s002.docx]

**Questionnaire**

**Section 1: Socio-demographic**

Please provide the following information by encircling and provide answer to the open-ended questions.

| S/N | Question | Answer | Remark |
| --- | --- | --- | --- |
| 101 | Age | ______ years |  |
| 102 | Gender | 1. Male 2. Female |  |
| 103 | Discipline | ______ |  |
| 104 | What is your program level? | 1. Master of Science/MPH 2. Specialty 3. Doctor of Philosophy 4. Sub-specialty |  |

**Section 2: Anxiety towards Research.**

To what extent do you agree or disagree with the following statements? Please rate your agreement on a scale ranging from 1 (Strongly Disagree) on the left-hand side to 7 (Strongly Agree) on the right-hand side.

| S/N | Items | Strongly Disagree | Disagree | Somewhat Disagree | Neutral | Somewhat Agree | Agree | Strongly Agree |
| --- | --- | --- | --- | --- | --- | --- | --- | --- |
| 201 | Postgraduate research makes me anxious. | 1 | 2 | 3 | 4 | 5 | 6 | 7 |
| 202 | Postgraduate research scares me. | 1 | 2 | 3 | 4 | 5 | 6 | 7 |
| 203 | A postgraduate research is stressful. | 1 | 2 | 3 | 4 | 5 | 6 | 7 |
| 204 | Postgraduate research is difficult. | 1 | 2 | 3 | 4 | 5 | 6 | 7 |

**Section 3: Factors Influencing Anxiety towards Research.**

To what extent do you agree or disagree with the following statements about **Self-Efficacy in Research**?

| S/N | Items | Strongly Disagree | Disagree | Somewhat Disagree | Neutral | Somewhat Agree | Agree | Strongly Agree |
| --- | --- | --- | --- | --- | --- | --- | --- | --- |
| 301 | I can realize the problems that may contribute to the field I work in. | 1 | 2 | 3 | 4 | 5 | 6 | 7 |
| 302 | I believe I am sufficient in creating hypotheses relevant to my research. | 1 | 2 | 3 | 4 | 5 | 6 | 7 |
| 303 | I can explain my research problem by drawing the necessary relations with prior research results. | 1 | 2 | 3 | 4 | 5 | 6 | 7 |
| 304 | I can find an appropriate title to my research. | 1 | 2 | 3 | 4 | 5 | 6 | 7 |
| 305 | I can effectively carry out the literature survey by using various channels (internet, library, etc.). | 1 | 2 | 3 | 4 | 5 | 6 | 7 |
| 306 | I systematically keep a record of the results of the literature survey. | 1 | 2 | 3 | 4 | 5 | 6 | 7 |
| 307 | I do not find it difficult at all to compare the results of my research to prior research results. | 1 | 2 | 3 | 4 | 5 | 6 | 7 |
| 308 | I can criticize the results of my research regarding research processes. | 1 | 2 | 3 | 4 | 5 | 6 | 7 |
| 309 | I can define the appropriate sampling method for my research. | 1 | 2 | 3 | 4 | 5 | 6 | 7 |
| 310 | I can decide which approaches to use for my research problem, be it quantitative or qualitative, or self-standing or incorporating various approaches together. | 1 | 2 | 3 | 4 | 5 | 6 | 7 |
| 311 | I can choose the appropriate data collection method necessary for my research. | 1 | 2 | 3 | 4 | 5 | 6 | 7 |
| 312 | I can test the validity and reliability of my research data through appropriate methods. | 1 | 2 | 3 | 4 | 5 | 6 | 7 |
| 313 | I can choose appropriate statistical methods to test or respond to my research hypotheses. | 1 | 2 | 3 | 4 | 5 | 6 | 7 |
| 314 | I can appropriately report on my analysis results. | 1 | 2 | 3 | 4 | 5 | 6 | 7 |
| 315 | I can discuss my research findings within a conceptual framework | 1 | 2 | 3 | 4 | 5 | 6 | 7 |
| 316 | I can create an appropriate titling system when writing up my research. | 1 | 2 | 3 | 4 | 5 | 6 | 7 |
| 317 | I can utilize appropriate referencing in my research, whether direct or indirect. | 1 | 2 | 3 | 4 | 5 | 6 | 7 |
| 318 | I can write an abstract for my research with ease. | 1 | 2 | 3 | 4 | 5 | 6 | 7 |

To what extent do you agree or disagree with the following statements about **Supervision Quality**? Please rate your agreement on a scale ranging from 1 (Strongly Disagree) to 5 (Strongly Agree).

| S/N | Items | Strongly Disagree | Disagree | Neutral | Agree | Strongly Agree |
| --- | --- | --- | --- | --- | --- | --- |
| 401 | Supervision is available when I need it. | 1 | 2 | 3 | 4 | 5 |
| 402 | My supervisor(s) makes a real effort to understand the difficulties I face. | 1 | 2 | 3 | 4 | 5 |
| 403 | My supervisor(s) provides me with additional information relevant to my topic. | 1 | 2 | 3 | 4 | 5 |
| 404 | I have given good guidance in topic selection and refinement. | 1 | 2 | 3 | 4 | 5 |
| 405 | My supervisor(s) provides helpful feedback on my progress. | 1 | 2 | 3 | 4 | 5 |
| 406 | I receive good guidance in my literature search | 1 | 2 | 3 | 4 | 5 |

To what extent do you agree or disagree with the following statements about **Research Infrastructure?**

| S/N | Items | Strongly Disagree | Disagree | Neutral | Agree | Strongly Agree |
| --- | --- | --- | --- | --- | --- | --- |
| 501 | I have access to a suitable working space. | 1 | 2 | 3 | 4 | 5 |
| 502 | I have good access to the technical support I need. | 1 | 2 | 3 | 4 | 5 |
| 503 | I have been able to organize good access to necessary equipment. | 1 | 2 | 3 | 4 | 5 |
| 504 | I have good access to computing facilities and services. | 1 | 2 | 3 | 4 | 5 |
| 505 | There is appropriate financial support for research activities. | 1 | 2 | 3 | 4 | 5 |

To what extent do you agree or disagree with the following statements about **Academic Support**?

| S/N | Items | Strongly Disagree | Disagree | Neutral | Agree | Strongly Agree |
| --- | --- | --- | --- | --- | --- | --- |
| 601 | In my academic department, there is a faculty member who cares about me. | 1 | 2 | 3 | 4 | 5 |
| 602 | In my academic department, there is a faculty member who is kind to me. | 1 | 2 | 3 | 4 | 5 |
| 603 | In my academic department, there is a faculty member who listens to me when I have something to say. | 1 | 2 | 3 | 4 | 5 |
| 604 | In my academic department, I have a peer who cares about me. | 1 | 2 | 3 | 4 | 5 |
| 605 | In my academic department, I have a peer who talks with me about my research challenges and ideas. | 1 | 2 | 3 | 4 | 5 |
| 606 | In my academic department, I have a peer who helps me when I have difficulties in my research. | 1 | 2 | 3 | 4 | 5 |
| 607 | My family helps me do well in my academic research endeavors. | 1 | 2 | 3 | 4 | 5 |
| 608 | My family motivates me to stay in my academic research pursuits. | 1 | 2 | 3 | 4 | 5 |
| 609 | My family is important in helping me to make my research plans and goals. | 1 | 2 | 3 | 4 | 5 |
| 610 | My family encourages me to continue further education and research beyond the postgraduate level. | 1 | 2 | 3 | 4 | 5 |
| 611 | My family can give me good advice about my academic and research journey. | 1 | 2 | 3 | 4 | 5 |
| 612 | My family cares about my academic and research pursuits. | 1 | 2 | 3 | 4 | 5 |

Once again, thank you for your time in completing the questionnaire! Your input is valuable for our research study!
