# Supplementary material for: Anxiety towards research and associated factors among postgraduate students of Jimma University Institute of Health, southwest Ethiopia
Source: PLOS Ment Health. 2026 Jul 2;3(7):e0000646. doi: 10.1371/journal.pmen.0000646 (PMC13327115; doi:10.1371/journal.pmen.0000646)
Supplement: S1 Table — (DOCX) [file pmen.0000646.s004.docx]

Reliability analysis of the tools

| **Measured variable** | **Measuring tool** | **Number of items** | **Cronbach’s alpha value** |
| --- | --- | --- | --- |
| Research anxiety level | Revised scale of students' attitudes toward research (R-ATR) | 4 | 0.950 |
| Research Self efficacy | Development of the research self-efficacy scale | 18 | 0.994 |
| Supervision Quality | Supervision Scale(SQ) | 6 | 0.924 |
| Research Infrastructure | Research Infrastructure Scale(RIS) | 5 | 0.913 |
| Acadamic Support | Perceived academic support questionnaire (PASQ) | 12 | 0.944 |
| Overall | | 49 | 0.920 |
