# Supplementary material for: Anxiety towards research and associated factors among postgraduate students of Jimma University Institute of Health, southwest Ethiopia
Source: PLOS Ment Health. 2026 Jul 2;3(7):e0000646. doi: 10.1371/journal.pmen.0000646 (PMC13327115; doi:10.1371/journal.pmen.0000646)
Supplement: S2 Table — (DOCX) [file pmen.0000646.s005.docx]

Sample size allocation for program level and disciplines.

| Program level | Population Size | Sample Size | Discipline | Population Size | Sample Size |
| --- | --- | --- | --- | --- | --- |
| Master of Science/MPH | 380 | 280 | Adult Health Nursing | 11 | 8 |
|  |  |  | Maternity Health Nursing | 12 | 9 |
|  |  |  | Clinical Pharmacy | 21 | 15 |
|  |  |  | Environmental Science and Technology | 10 | 7 |
|  |  |  | Epidemiology | 12 | 9 |
|  |  |  | General Public Health | 10 | 7 |
|  |  |  | Health Promotion and Health Behavior | 7 | 5 |
|  |  |  | Medical Microbiology | 16 | 12 |
|  |  |  | Medical Physiology | 11 | 8 |
|  |  |  | Reproductive Health | 10 | 7 |
|  |  |  | Health Monitoring and Evaluation | 16 | 12 |
|  |  |  | Pharmaceutical supply Chain management | 17 | 13 |
|  |  |  | Human Nutrition | 11 | 8 |
|  |  |  | Integrated Clinical and Community Mental Health | 21 | 15 |
|  |  |  | Medical Parasitology | 10 | 7 |
|  |  |  | Field Epidemiology | 7 | 5 |
|  |  |  | Pharmaceutical Quality Assurance and Regulatory Affairs | 13 | 10 |
|  |  |  | Environmental Health Science | 6 | 4 |
|  |  |  | Clinical Laboratory Sciences Specialty in Clinical Chemistry | 13 | 10 |
|  |  |  | Clinical Laboratory Sciences Specialty in Hematology and Immunohematology | 7 | 5 |
|  |  |  | Clinical Anatomy | 5 | 4 |
|  |  |  | Clinical Laboratory Sciences Specialty Laboratory Management | 6 | 4 |
|  |  |  | Medical Biochemistry | 6 | 4 |
|  |  |  | Clinical Anesthesia | 17 | 13 |
|  |  |  | Bioinformatics | 5 | 4 |
|  |  |  | Clinical Midwifery | 21 | 15 |
|  |  |  | Midwifery Education | 8 | 6 |
|  |  |  | Child and Pediatrics Nursing | 10 | 7 |
|  |  |  | Health Economics | 9 | 7 |
|  |  |  | Health Systems Management | 10 | 7 |
|  |  |  | Neonatal Health Nursing | 7 | 5 |
|  |  |  | Emergency and Critical Care Nursing | 9 | 7 |
|  |  |  | Psychiatric and Mental Health Nursing | 6 | 4 |
|  |  |  | Pharmacology | 5 | 4 |
|  |  |  | Pharmaceutics | 8 | 6 |
|  |  |  | Dietetics | 7 | 5 |
| Specialty | 140 | 103 | Pediatrics and Child Health | 12 | 9 |
|  |  |  | Obstetrics And Gynecology | 18 | 14 |
|  |  |  | Internal Medicine | 14 | 10 |
|  |  |  | Ophthalmology | 15 | 11 |
|  |  |  | Surgery | 17 | 13 |
|  |  |  | Pathology | 5 | 4 |
|  |  |  | Anesthesiology, Critical Care, And Pain Medicine | 24 | 18 |
|  |  |  | Psychiatry | 11 | 8 |
|  |  |  | Emergency and Critical Care Medicine | 18 | 13 |
|  |  |  | Radiology | 6 | 4 |
| Doctor of Philosophy | 40 | 29 | Pharmaceutical Sciences | 2 | 1 |
|  |  |  | Health communication and Health Behavior | 9 | 7 |
|  |  |  | Evidence-Based Health Care | 4 | 3 |
|  |  |  | Applied Ecology | 4 | 3 |
|  |  |  | Environmental Health | 6 | 4 |
|  |  |  | Tropical and Infectious Diseases | 4 | 2 |
|  |  |  | Medical Microbiology | 1 | 1 |
|  |  |  | Health System and Policy | 5 | 4 |
|  |  |  | Public Health - Reproductive Health | 1 | 1 |
|  |  |  | Medical Physiology | 1 | 1 |
|  |  |  | Human Nutrition | 3 | 2 |
| Subspecialty | 13 | 10 | Gynecological Oncology | 5 | 4 |
|  |  |  | Pediatrics Hematology Oncology | 3 | 2 |
|  |  |  | Urogynaecology | 2 | 2 |
|  |  |  | Gastrointestinal Cancer Surgery | 3 | 2 |
