# Supplementary material for: Anxiety towards research and associated factors among postgraduate students of Jimma University Institute of Health, southwest Ethiopia
Source: PLOS Ment Health. 2026 Jul 2;3(7):e0000646. doi: 10.1371/journal.pmen.0000646 (PMC13327115; doi:10.1371/journal.pmen.0000646)
Supplement: S3 Table — (DOCX) [file pmen.0000646.s006.docx]

Multicollinearity assessment using Variance Inflation Factor (VIF)

| **Dependent Variable** | **Independent variables** | **Collinearity Statistics** | |
| --- | --- | --- | --- |
|  |  | Tolerance | VIF |
| Research anxiety level | Gender | .899 | 1.112 |
|  | Program level | .803 | 1.245 |
|  | Research Self efficacy | .653 | 1.530 |
|  | Supervision Quality | .712 | 1.405 |
|  | Research Infrastructure | .829 | 1.206 |
|  | Acadamic Support | .689 | 1.451 |
