# Supplementary material for: Anxiety towards research and associated factors among postgraduate students of Jimma University Institute of Health, southwest Ethiopia
Source: PLOS Ment Health. 2026 Jul 2;3(7):e0000646. doi: 10.1371/journal.pmen.0000646 (PMC13327115; doi:10.1371/journal.pmen.0000646)
Supplement: S4 Table — (DOCX) [file pmen.0000646.s007.docx]

Average, median and SD statistics of variables.

| **Variable** | **Mean** | **Median (Cut-point in this study)** | **SD** |
| --- | --- | --- | --- |
| Research anxiety level | 4.3563 | 4.5200 | 1.48502 |
| Research Self efficacy | 4.9876 | 5.3889 | 1.52870 |
| Supervision Quality | 3.4648 | 3.5000 | 1.03839 |
| Research Infrastructure | 3.1214 | 3.0000 | 1.06392 |
| Acadamic Support | 3.4224 | 3.4167 | .87871 |
